# Anti-Inflammation Activities of Mycosporine-Like Amino Acids (MAAs) in Response to UV Radiation Suggest Potential Anti-Skin Aging Activity

**DOI:** 10.3390/md12105174

**Published:** 2014-10-14

**Authors:** Sung-Suk Suh, Jinik Hwang, Mirye Park, Hyo Hyun Seo, Hyoung-Shik Kim, Jeong Hun Lee, Sang Hyun Moh, Taek-Kyun Lee

**Affiliations:** 1 South Sea Environment Research Department, Korea Institute of Ocean Science and Technology, Geoje 656-830, Korea; E-Mails: sung-suk.suh@kiost.ac (S.-S.S.); jinike12@kiost.ac (J.H.); mirye@kiost.ac (M.P.); 2 Anti-Aging Research Institute of Bio-FD&C Co. Ltd, Incheon 406-840, Korea; E-Mails: hhseo@biofdnc.com (H.H.S.); hskim@biofdnc.com (H.-S.K.); jhlee@biofdnc.com (J.H.L.)

**Keywords:** mycosporine-like amino acids (MAAs), HPLC, mycosporine-glycine, shinorine, porphyra-334

## Abstract

Certain photosynthetic marine organisms have evolved mechanisms to counteract UV-radiation by synthesizing UV-absorbing compounds, such as mycosporine-like amino acids (MAAs). In this study, MAAs were separated from the extracts of marine green alga *Chlamydomonas hedleyi* using HPLC and were identified as porphyra-334, shinorine, and mycosporine-glycine (mycosporine-Gly), based on their retention times and maximum absorption wavelengths. Furthermore, their structures were confirmed by triple quadrupole MS/MS. Their roles as UV-absorbing compounds were investigated in the human fibroblast cell line HaCaT by analyzing the expression levels of genes associated with antioxidant activity, inflammation, and skin aging in response to UV irradiation. The mycosporine-Gly extract, but not the other MAAs, had strong antioxidant activity in the 2,2-diphenyl-1-picryhydrazyl (DPPH) assay. Furthermore, treatment with mycosporine-Gly resulted in a significant decrease in COX-2 mRNA levels, which are typically increased in response to inflammation in the skin, in a concentration-dependent manner. Additionally, in the presence of MAAs, the UV-suppressed genes, procollagen C proteinase enhancer (PCOLCE) and elastin, which are related to skin aging, had increased expression levels equal to those in UV-mock treated cells. Interestingly, the increased expression of involucrin after UV exposure was suppressed by treatment with the MAAs mycosporine-Gly and shinorine, but not porphyra-334. This is the first report investigating the biological activities of microalgae-derived MAAs in human cells.

## 1. Introduction

The increased solar ultraviolet radiation (UV, 280–400 nm) reaching the Earth’s surface due to the depletion of the stratospheric ozone has significant effects on the cellular metabolism of living organisms [1,2,3]. For example, harmful doses of UV radiation can penetrate deep into a water column, influencing photosynthetic marine organisms, such as cyanobacteria, phytoplankton, and microalgae, directly causing cell damage by affecting the stability of DNA and indirectly by producing reactive oxygen species [4,5]. It may have detrimental effects on major physiological and biochemical processes, including survival, cell growth, pigmentation, and photosynthetic oxygen production [6,7,8]. Chronic exposure to ultraviolet (UV) radiation, which is typically divided into wavelength ranges as UVA (315–400 nm), UVB (280–315 nm), and UVC (100–280 nm), induces changes in skin structures in humans, referred to as photoaging, which covers epidermal atrophy, an increase in melanocyte numbers, heavy deposition of dystrophic and truncated elastic fibers in the dermis, a decrease in the number of collagen fibers, and the presence of dermal inflammatory infiltrates, leading to significant changes in the expression levels of photoaging-associated genes. For example, UV exposure profoundly influences skin aging through the destruction of collagen, a major contributor to the loss of skin suppleness, and reduction of elastin content in the extracellular matrix, which is a collagen-binding molecule [9]. It was recently reported that a component of green algae has potential protective effects by preventing UVB-suppressed elastin and pro-collagen gene expression, and may protect against UVB irradiation-induced skin damage [10]. Procollagen C proteinase enhancer (PCOLCE) is an important determinate of procollagen processing in the regulation of collagen deposition in the skin [11]. In addition, a keratinocyte molecule, involucrin, acts as a marker of keratinocyte differentiation and its expression is increased by UV exposure [12,13]. It has been reported that microalgae extracts might protect skin through the inhibition of UV radiation-induced upregulation of genes, including involucrin [14]. In UV-induced inflammation, the pro-inflammatory gene, COX-2, is highly induced. Interestingly, many seaweed extracts exhibit significant biological activities, including anti-inflammatory activities, by the suppression of COX-2 in response to UV radiation [15].

Many photosynthetic marine organisms, which are exposed to UV radiation, have evolved tolerance mechanisms to minimize its negative effects, including DNA repair systems, radical quenchers, and antioxidants [16,17,18]. One of the most important of these mechanisms is the synthesis of UV-absorbing compounds, mycosporine-like amino acids (MAAs), which are characterized by a cyclohexenone or cyclohexenimine chromophore conjugated to the nitrogen substituent of an amino acid, with absorption maxima ranging from 310 to 360 nm [19,20]. The MAA family currently consists of ~20 members, including mycosporine-glycine, palythine, palythinol, asterina-330, porphyra-334, and shinorine. Porphyra-334 and shinorine were first found in the marine red algae *Porphyra tenera* and *Chondrus yendoi*, respectively, and later in many marine red algal species [21,22]. Shinorine is the most frequently occurring and abundant MAA in many microalgae species [23]. Mycosporine-glycine was first isolated from the zoanthidian *Palythoa tuberculosa* [24], and is not only one of the most abundant MAAs, identified in a broad array of marine species, but also shows reasonably strong antioxidant activity [25]. The ability of MAAs to absorb UV radiation and dissipate its energy without the formation of reactive oxygen species protects microalgae from UV damage [26]. In fact, it has been reported that a sun screen containing MAAs from red algae can protect the skin from the harmful effects of UVA [27]. Although the primary synthesis of MAAs is in response to UV, MAAs function in several roles in addition to UV protection. For example, the oxo-carbonyl mycosporines and porphyra-334 have antioxidant activity and prevent cellular damage resulting from UV-induced production of reactive oxygen species, leading to MAA synthesis in response to high levels of photosynthetically active radiation [28]. Several standard *in vitro* assays, including the scavenging potential for hydrosoluble radicals, antioxidant activity in a lipid medium, and the scavenging capacity for superoxide radicals, have been performed to determine the potential antioxidant capabilities of purified aqueous extracts of mycosporine-like amino acids [29,30].

Recently, the biochemical and phylogenetic relationship between the gene counterparts involved in MAA biosynthesis via either the shikimate or pentose-phosphate pathway was revealed using molecular approaches, such as a transcriptome mining analysis [31]. However, despite the numerous ecological and physiological studies on MAAs and their protection against UV damage in marine organisms, including microalgae, our understanding of the role of MAAs at the molecular level is still poor. In this study, we investigated the physiological and molecular characteristics of various MAAs derived from the green alga *Chlamydomonas hedleyi* in terms of the intrinsic function of MAAs in response to UV radiation.

## 2. Results and Discussion

### 2.1. Identification of MAAs from Chlamydomonas hedleyi by HPLC Assay

**Figure 1:**
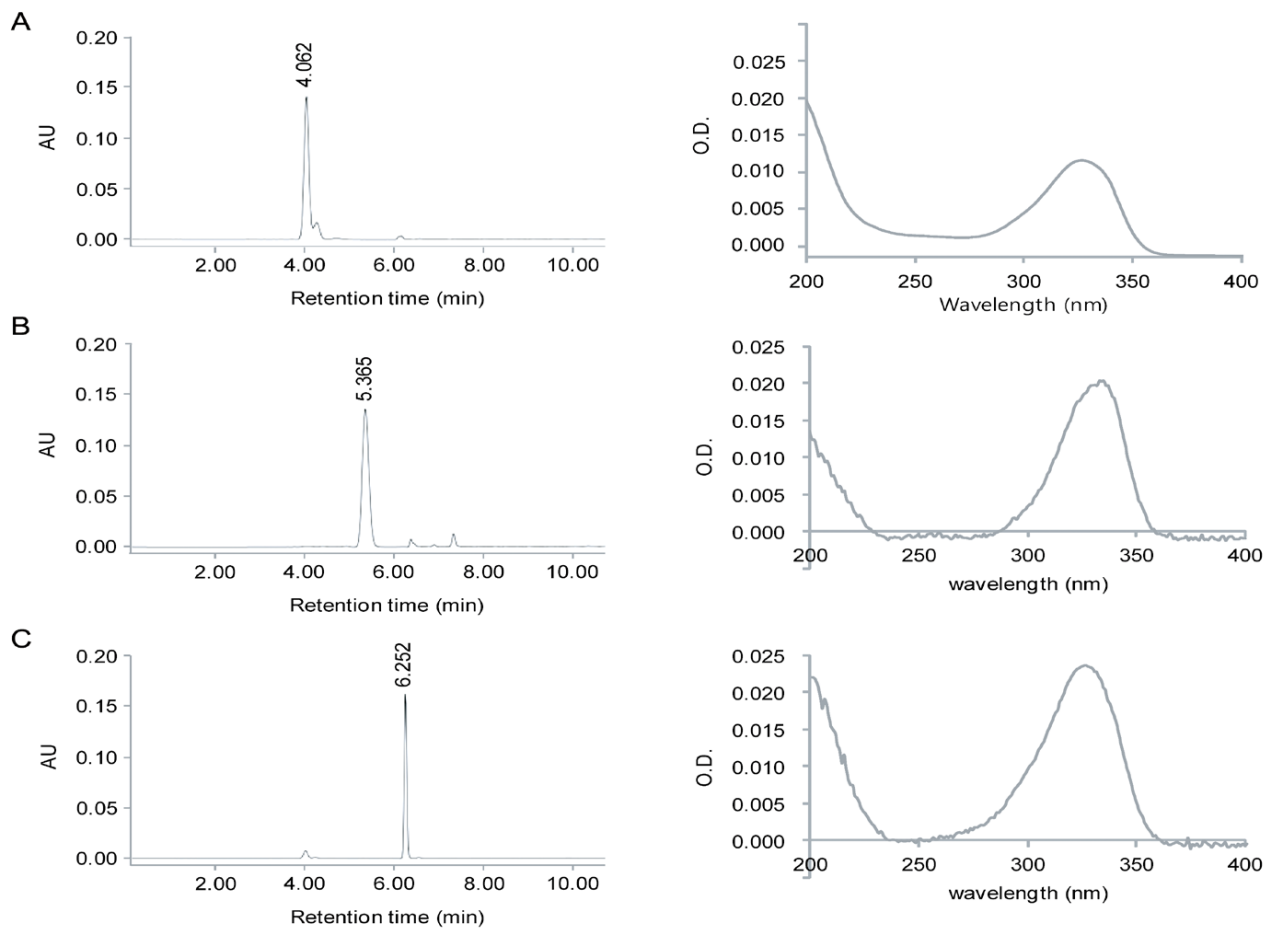
(**A**) HPLC chromatogram and absorption spectra of purified mycosporine-Gly (**A**); porphyra-334 (**B**); and shinorine (**C**) from *Chlamydomonas hedleyi*.

**Figure 2:**
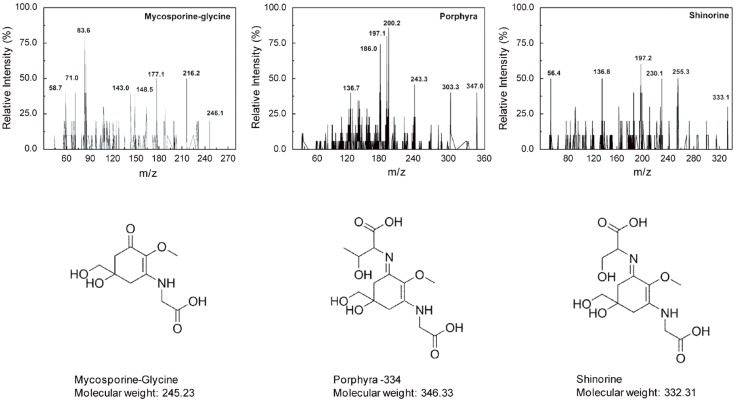
MS/MS analysis of mycosporine-Gly, shinorine, and porphyra-334 from *Chlamydomonas hedleyi*.

The MAAs, porphyra-334, shinorine, and mycosporine-Gly, extracted from the microalga *Chlamydomonas hedleyi*, were identified by comparing their retention times (RT) during HPLC separation as well as their characteristic UV absorption spectra *via* diode array detection (DAD) (Figure 1). Due to the lack of fine spectral absorption, the only spectral characteristic available for MAA identification is the position of the absorption maximum (λ_max_). In the UV-Vis spectra, there is a pronounced increase in the absorption in the near UV region, at ~334 nm (Figure 1). Furthermore, the molar absorption coefficients of each UV-absorption peak of 2.82 × 10^4^, 4.46 × 10^4^, and 4.21 × 10^4^ M^−1^·cm^−1^ corresponded to mycosporine-Gly, shinorine, and porphyra-334, respectively. These differences in spectral characteristics and the value of their extinction coefficient (ε) spectra are due mainly to variation in the attached side groups and nitrogen substitutes of the amino cyclohexenimine ring. The HPLC spectrum had three prominent peaks with retention times of 4.060, 4.299, and 6.335 min for mycosporine-Gly, shinorine, and porphyra-334, respectively (Figure 1), indicating that these compounds were in the mixture of purified MAAs. Mass spectrometry detection (HPLC/MS), using pure reference compounds, is invaluable in the identification of MAAs because of its high sensitivity and tandem mass spectrometric technology. The positive ESI mass spectral fragmentation patterns of each MAA, as obtained by triple quadrupole MS/MS (Figure 2), exhibited many distinctive features. The fragmentation patterns are as expected for mycosporine-Gly, shinorine, and porphyra-334. Additionally, the concentrations of porphyra-334, shinorine, and mycosporine-Gly were 0.12, 0.17, and 0.32 μmol·g^−1^, respectively.

### 2.2. Antioxidant Activity of MAAs with Free Radical-Trapping Abilities

**Figure 3:**
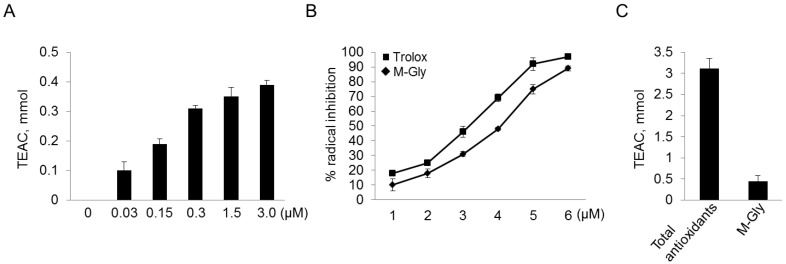
Antioxidant capacities of mycosporine-Gly (M-Gly), were determined using the Trolox equivalent antioxidant capacity (TEAC). (**A**,**B**) Antioxidant activities at the different concentrations of M-Gly and measurement of IC_50, _respectively; (**C**) Antioxidant capacities of total water-soluble antioxidants and M-Gly. IC_50_ values calculated denote the concentration of the sample required to decrease the absorbance by 50%. Trolox was used as a positive control.

To investigate the efficacy of MAAs as a potential sunscreen, their radical-scavenging capacities were measured. Decolorization of DPPH was monitored both visually and using a spectrophotometer. Figure 3 shows the percentage antioxidant activity detected using various concentrations of purified MAAs. Among the three MAAs, the radical-scavenging activity of mycosporine-Gly increased with increasing concentration up to 1.5 mM (Figure 3A). The results indicate that mycosporine-Gly may act as a strong antioxidant and so prevent cellular damage as a result of the UV-induced production of free radicals. The antioxidant activity of mycosporine-Gly is considerably lower than that of ascorbic acid, which was used as the positive control. Consistent with a previous report [25], we found that mycosporine-Gly had antioxidant activity, but the other MAAs did not. This may be due to the fact that oxo-carbonyl MAAs, including mycosporine-Gly, have moderate antioxidant activity, whereas the imino-MAAs, which have a cyclohexenimine core (porphyra-334 and shinorine), do not have greater antioxidant activity than the negative control group. This indicates that mycosporine-Gly was decomposed, whereas porphyra-334 and shinorine were stable. A possible reason for the increased oxidation of imino-MAAs may be the heat needed to dissipate the absorbed energy [32,33]. In fact, heat often accelerates the oxidation process, because temperature can function as a type of activation energy. If the reaction systems associated with the oxidative process inside the organism do not contain enough energy to overcome the threshold, no oxidative reaction will occur. In addition, the coral *Sylophora pistillata*, which contains only small amounts of mycosporine-Gly, is significantly more sensitive to heat-induced oxidative stress than *Platygyta ryukyuensis*, which has a 20-fold higher concentration of mycosporine-Gly [34]. Thus, the heat energy derived from UV absorption by MAAs in *Chlamydomonas hedleyi* may be sufficient to induce and accelerate the oxidative process, leading to increased oxidative activities in a shinorine-concentration-dependent manner. In addition, consistent with previous study [35], mycosporine-Gly showed a high IC_50_ value (IC_50_ = 4.23 ± 0.21) that was comparable to that of ascorbic acid (IC_50_ = 3.12 ± 0.18) (Figure 3B). In particular, mycosporine-Gly contributed approximately 15% of the total water-soluble scavenging activity in the water extract (Figure 3C).

Our data disagree with a previous report that porphyra-334 and shinorine had antioxidant activities [32,35,36]. This may be due to the use of different substrates or experimental conditions in the measurement of antioxidant activity. For example, porpyra-334 and shinorine exhibited antioxidant activity against oxidation induced by lipid peroxidation, but had only low hydrosoluble free-radical-scavenging activity [35,36]. Furthermore, with regard to the DPPH-radical-scavenging activities of porphyra-334, heat treatment may be necessary to achieve antioxidant activity, which otherwise is very low [32].

### 2.3. Role of MAAs in Skin Inflammation and Aging

**Figure 4:**
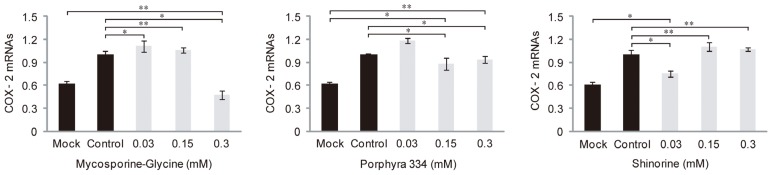
Expression levels of COX2 mRNAs in response to different concentrations of mycosporine-like amino acids (mycosporine-glycine, porphyra 334, and shinorine) under UV radiation. The error bars indicate standard deviations (means ± SD, *n* = 6). One-way ANOVA, *p <* 0.001, *F* (4, 25) = 19.63 in mycosporine-glycine, *p <* 0.01, *F* (4, 25) = 31.91 in porphyra 334, and *p <* 0.01, *F* (4, 25) = 21.03 in shinorine; *Post hoc* Tukey test: * *p* < 0.05 and ** *p* < 0.001.

**Figure 5:**
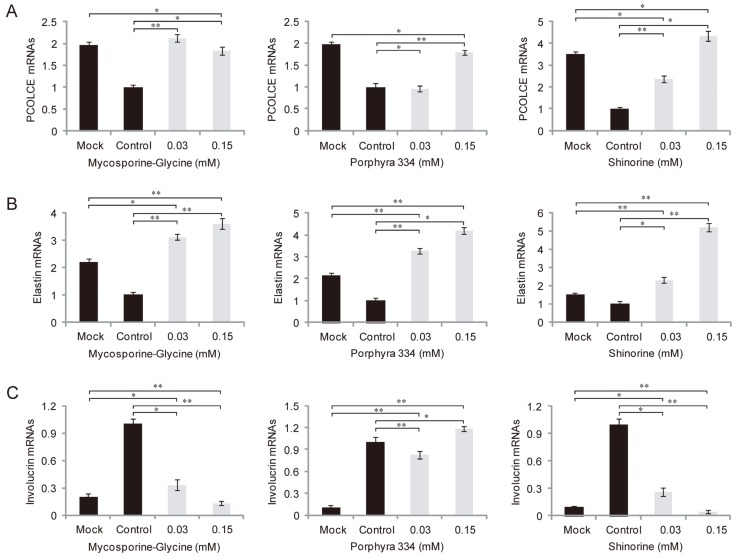
Expression levels of procollagen C proteinase enhancer (PCOLCE) (**A**); elastin (**B**); and involucrin (**C**) mRNAs in response to different concentrations of MAAs under UV radiation. The error bars indicate standard deviations (means ± SD, *n* = 6). One-way ANOVA (*p <* 0.05); *Post hoc* Tukey test: * *p <* 0.01 and ** *p <* 0.001.

We examined whether microalgae-derived MAAs had anti-inflammatory activity against UV irradiation. HaCaT cells, immortalized human keratinocytes, were treated with 0.03, 0.15, or 0.3 mM MAAs plus UV irradiation. qRT-PCR was used to determine the mRNA levels of the COX2 gene, which plays a key role in the generation of inflammatory responses and the expression of which is increased in inflamed tissue. UV-induced COX-2 expression decreased to that of the control UV-mock treated cells in the presence of a high concentration of mycosporine-Gly (0.3 mM), but not lower concentrations (0.03 and 0.15 mM), while expression was decreased by only the lowest concentrations of shinorine (0.03 mM) (Figure 4). However, porphyra-334 had no effect on COX2 expression at any concentration tested. Together, these data suggest that mycosporine-Gly and shinorine inhibited inflammation caused by UV radiation through modulation of COX-2 expression. Next, to evaluate the effects of MAAs on skin aging influenced by UV, we investigated the expression pattern of related genes in samples treated with both UV and MAAs. The expression levels of procollagen c-endopeptidase enhancer (PCOLCE), which binds to procollagen and enhances procollagen c-proteinase activity, and elastin mRNAs were strongly suppressed after UV irradiation (Figure 5A,B), whereas that of involucrin was elevated (Figure 5C). In the presence of MAAs, UV-suppressed levels of PCOLCE and elastin rebounded to those in UV-mock treated cells (*i.e.*, were more highly expressed; Figure 5A,B). In contrast, the involucrin mRNA level was downregulated, except when porphyra-334 was applied (Figure 5C). When treated with both porphyra-334 and shinorine, PCOLCE expression was increased in a concentration-dependent manner (Figure 5A). Similar expression patterns were observed for elastin after treatment with mycosporine-Gly, porphyra-334, and shinorine (Figure 5B). Interestingly, elastin was more highly expressed in cells treated with MAAs and UV irradiation than in UV-mock treated cells, suggesting that MAAs modulate elastin expression via as-yet unknown regulatory mechanism(s).

In addition to the antioxidant activity of mycosporine-Gly, it may also protect against the skin inflammation caused by UV through the modulation of inflammation-related genes, including COX-2. The level of COX-2 mRNAs decreased in response to mycosporine-Gly, but not the other MAAs. Interestingly, at the same concentration of mycosporine-Gly (0.3 mM), antioxidant activity was high and UV-elevated COX-2 expression was simultaneously suppressed, suggesting that the regulation of COX-2 may be related to the oxidative process in which mycosporine-Gly is involved. We also found that the three MAAs from *Chlamydomonas hedleyi* act as anti-aging factors by modulating the expression of genes associated with aging in the skin, such as PCOLCE, elastin, and involucrin. It has also been found that they had a promoting effect on the proliferation of human cells [37]. UV radiation influences skin aging through the production of reactive oxygen species, leading to oxidative alteration of cell constituents and DNA mutation [4,5]. In this study, although only mycosporine-Gly exhibited antioxidant activity, UV-modulated expression of aging-associated genes was affected by mycosporine-Gly, porphyra-334, and shinorine. This indicates that in addition to oxidant stress, other mechanisms modulate the expression of aging-associated genes.

## 3. Experimental Section

### 3.1. Growth of Chlamydomonas hedleyi

*Chlamydomonas hedleyi* strain KMMCC 118 was obtained from the Korean Marine Microalgae Culture Center (Busan, Korea). This strain was sampled from the South Sea (34°45′ N, 128°40′18ʺ E) and deposited in the Korean Marine Microalgae Culture Center. It was identified using the 18S rRNA gene and its GenBank accession number is JQ315503. *Chlamydomonas hedleyi* was grown in a 500-mL Erlenmeyer flask containing 250 mL of f/2 medium to an initial cell density of 5 × 10^4^ cells·mL^−1^ and suspended in a thermoregulated aquarium at a temperature and light regime of 22 ± 1 °C/80 μmol photon·m^−2^·s^−1 ^under a 16:8 h light:dark cycle on a rotary shaker (180 rev·min^‑1^). The pH of the medium ranged from 8 to 9, maintained by sparging CO_2_ (l%)-enriched air through the culture. After cells were grown for 7 days, 0.25 L of culture medium was harvested for dry weight biomass. In the culture system, dry weight (DW) and crude MAAs accounted for ~3.72 g/L and ~0.01 mg/g DW, respectively, as described previously [38].

### 3.2. Identification and Characterization of MAAs

MAAs were assayed as described previously [39]. Briefly, triplicate samples of dried alga (20 mg DW) were extracted for 2 h in screw-capped centrifuge vials filled with 1-mL 20% aqueous methanol (v/v) at 45 °C. After centrifugation (5000 *g*, 10 min, room temperature), 700 μL of the supernatant were evaporated to dryness under vacuum at 45 °C (Jouan evaporator centrifuge RC 10.09, Cedex, France) and the residue was redissolved in 500 μL of sterile distilled water followed by the addition of 100 μL chloroform with gentle vortexing. After centrifugation (10,000 *g*, 5 min), the uppermost water phase was transferred carefully into new microcentrifuge tubes to remove contaminating lipophilic photosynthetic pigments from water-soluble MAAs. Finally, the solvent was evaporated and the sample was taken up in 50% methanol for further analysis.

All samples were analyzed quantitatively by HPLC (Shimadzu-LC20A, Seattle, WA, USA) with a DAD-SPD M20A detector (Quantum Northwest, Seattle, WA, USA), according to a previous report [40], with a slight modification in the pH of the mobile phase. Thus, aliquots (30–60 μL) of the extract were diluted in 100% HPLC-grade methanol to a final volume of 0.5 mL and dried in a rotary evaporator. The residues were re-dissolved in 600 μL of water and then diluted (1:10) with a pH 3.00 solution of aqueous trifluoroacetic acid 0.2% and ammonium hydroxide. The final solution was ultra-filtered with a Whatman 100 kDa filter (12,000 *g*, 20 min) to remove water-insoluble materials and large molecules. Then, 10 μL samples of the resulting solution were injected into the HPLC system at a 1 mL·min^−1^ flow rate. The signals were processed with the Class-VP software (Quantum Northwest, Seattle, WA, USA). Identification of MAAs was accomplished by their absorption maxima and retention times, calibrated with authenticated standards of: shinorine, mycosporine-2-glycine, palythine-serine, palythine, prophyra-334, mycosporine-methylamine-serine, mycosporine-glycine, palythinol and mycosporine-methylamine-threonine [41], which were provided by F. Figueroa, University of Malaga (Malaga, Spain). We ran the HPLC/MS of each MAA separately.

### 3.3. MS/MS Analysis

Dried MAAs extracts were dissolved in deionized water (1 mg/mL); a final concentration of 100 ppm was achieved using 50% methanol with 0.1% formic acid and passing it through a 0.45-μm membrane filter. Analyses of MAAs were performed on an ESI-MS/MS system (Thermo Fisher Scientific, San Jose, CA, USA) consisting of an AB SCIEX 3200 QTRAP MS/MS (Applied Biosystems, Foster City, CA, USA) with an ESI source (TurbolonSpray, Applied Biosystem/MDS SCIEX, Concord, Canada). Data acquisition and processing were performed with the AB SCIEX Analyst 1.5 software (AB SCIEX Korea Limited Company, Seoul, Korea). All MAAs were quantified in Product Ion (MS2) mode in positive mode. The syringe pump method properties (Tune Control) were as follows: syringe diameter 4.6 mm and flow rate 10.00 μL/min. The optimal ESI source conditions were as follows: turbo heater temperature (TEM) 300 °C, ion spray voltage 5500 V, curtain gas 10 psi, nebulizing gas (gas 1) 15 psi and heated gas (gas 2) 40 psi. The collision energy (CE) and entrance potential (EP) were set separately at 35 V and 4.50 V; the mass transition of MAAs, optimal declustering potential (DP) 51 V and collision cell exit potential (CXP) 4 V.

### 3.4. Assay for Antioxidant Activity

The DPPH-free radical scavenging capacity of purified MAAs was evaluated as described previously [42]. Briefly, a 0.2-mL dose of the tested seed extracts, including different concentrations (0.03, 0.15, 0.3, 1.5, 3.0 mM; in methanol) of MAAs, was added to a 3.8-mL ethanol solution of the DPPH radical (final concentration, 0.1 mM). The mixture was shaken vigorously for 1 min by vortexing and left to stand at room temperature in the dark for 30 min. The absorbance of the sample (A_sample_) was measured using a UV 160 spectrophotometer at 517 nm against an ethanol blank. A negative control (A_control_) was taken after adding DPPH solution to 0.2 mL of the respective extraction solvent. The percent of DPPH discoloration in the sample was calculated according to the equation: % discoloration = [1 − (A_sample_/A_control_)] × 100. Additionally, ascorbic acid was used as a positive control because it functions as an effective antioxidant in reactions with aqueous peroxyl radicals.

### 3.5. Cell Culture

Keratinocytes account for 90% of all skin epidermal cells, the stratified squamous epithelium forming the outer layer of the skin, where many skin diseases originate. The human keratinocyte cell line, HaCaT, was purchased from the American Type Culture Collection (ATCC, Manassas, VA, USA). Cells were cultured in Dulbecco’s modified Eagle’s medium (DMEM) containing 10% heat-inactivated FBS and 2 mM l-glutamine and 100 U·mL^−1^ penicillin‑streptomycin in a humidified atmosphere. For cell culture maintenance, the medium was changed every 2–3 days and cells were split at 80% confluence with trypsinization. For the experiment, cells were seeded at an optimal density of 14 × 10^3^/cm^2^. For exposure to UV, cells were seeded in a 24-well plate at 0.1 × 10^6^/well and grown overnight before UV treatment.

### 3.6. UV Exposure Procedures

UV exposure was performed as described previously [42]. A Philips Original Home Solarium sun lamp (model HB 406/A; Philips, Grogningen, Holland) equipped with a UV lamp delivering a flux of 23 mW/cm^2^ between 300 and 400 nm was used as a UV radiation source at a distance of 20 cm from the samples. The dose of UV received from above by the samples was measured using a UV Power Pack Radiometer (EIT Inc., Sterling, VA, USA), while the emission spectrum was assessed using a StellarNet portable spectroradiometer (Tampa, FL, USA). Cells grown on a 24-well culture plate for UV irradiation were first washed with phosphate-buffered saline (PBS) and covered with a thin layer of PBS. Various concentrations (0, 0.03, 0.15, and 0.3 mM) of each MAA were added on top of the wells prior to irradiation. Cells were irradiated for 15 min (275 kJ/m^2^). This UV dose is equivalent to ~90 min of sunshine on the French Riviera (Nice, French) in the summer at noon [43].

### 3.7. RNA Extraction and qRT-PCR

Total RNA was extracted using the TRIzol reagent (Invitrogen, cat. # 15596-018, Carlsbad, CA, USA) following the manufacturer’s protocol. At the end of the extraction, the isolated RNA was dissolved in 35 μL of RNase-free water and incubated for 10 min at 55 °C. An aliquot of RNA (5 μg) was then used for cDNA synthesis using the SuperScript first-strand cDNA synthesis kit (Invitrogen, Carlsbad, CA, USA). RT-PCR was performed using the Rotor Gene Q Real-time PCR Machine (Qiagen, Valencia, CA, USA) with QuantiTect primer assays (COX-2: QT00040586, PCOLCE: QT01005725, elastin: QT00034594, involucrin: QT00082586). The housekeeping gene, 18S rRNA (QT00199367), was used for normalization. qRT-PCR was performed for 35 cycles under the following conditions: denaturation (15 s, 94 °C), annealing (30 s, 55 °C), and extension (30 s, 72 °C).

### 3.8. Statistical Analysis

Mean values and their standard deviations were calculated from six biological replicates. The statistical significance of the difference between means was tested using one-way ANOVA followed by a Tukey B multi-range test; *p* values <0.05 were considered to indicate statistical significance.

## 4. Conclusions

In conclusion, the microalga *Chlamydomonas hedleyi* contains UV-absorbing MAAs, which may provide protection to the skin against the impact of UV radiation. In particular, MAAs act as UV-absorbing compounds, modulating the expression of genes associated with oxidative stress, inflammation, and skin aging caused by UV. Our data provide new insight into the use of MAAs in the industrial and pharmacological development of biological sunscreens and antioxidants.
